# 

**Published:** 1837

**Authors:** 


### INDEX.

A.

PAGE.

Absorption of Placenta, 583
Acetate of Lead in Dysentery, 19
Aconite effects of in animals. 262
Alum in Typhoid fevers, 267
Aluminia in Cholera, 268
Amawrosis from Lead colic, 111
Anatomy of Eye, 424
Appointment, 480
Artificial joint, 633
pupil, 107, 144
Arteries of inflamed parts, 590
Ascitic effusion, 343
Asphyxia, recovery from, 282
Atmospheric heat in wounds, 568

B.

Bals. Copain, mode of administration, 431
Belladonna, in retention of urine, 566
Bleeding in chill of intermittents, 135, 349
Borate of Sodae in dysmenorrhœa, 508
Brain and nerves, Experiments on 587
British and Foreign Review, 300

C.

Caesarean operation, 13, 574
Carcinoma of Tongue 255
Cataract, 144
Case of Dr. Crosby, 255
Causes of motion of blood in capillary vessels, 591
Cerebro-Spinal nerves, 562
Cincinnati College, 319
Cincinnati Eye Infirmary, 479
Hospital, 321
Medical Society, 168
Health of, 167
Climate of Madeira, 131
Cloride of Sodium in fever, 146
Cold baths in chorea, 257
water, a stimulus, 137

PAGE

applications, 280
dash in intermittents, 519
Comparative physiology, 350
Consultation with Quacks, 293
Convulsions, Extraordinary case of 636
in children, 477
Concealed Strangulated Hernia, 631
Consultation Letters, 643
Creosote, 305
action of 154
Medical properties of, 466
employment of, 155
Mode of administration, 157
Croton Tiglium, 105
Cyclopædia of Practical Medicine, 383
of Medicine and Surgery, 414

D.

Defloration, 53
Delivery, 66
Diary of an old physician, 169, 500
Delirium Tremens, 146
Diseases of antrum, 333,493
Discourses and Lectures, 644
Doctor Quacks, 613
Double vision, 258
Doubtful sex, 52

E.

Editorial note, 491
Effects of medicines on heart, 266
Electricity and Galvanism in paralysis, 580
Elementary forms of disease, 564
Elephantrasis of the Arabians, 279
Enlarged tonsils, cure of, 112
Epidemic erysipelas, 225
Exchange Journals, 165
Experiments and observations on coecum, 395

F

Family medicines, 617
Firschi's craniology, 257

PAGE
Fungous Haematodes, 307
Fumigations in hooping cough, 270
Fracture of cervical vertebra, 328
Fractures of extremities, 107

G.

Galens pills and plasters, 617
Genuine specific ointment, 619
Goitre, Extirpation of 117

H.

Hæmoptysis covered, 112
Health and disease in communities, 86
Hermaphrodism, 52
Horse Doctor's Advertisement, 620
Hospital in Paris 96
In the West, 323
Their Regulations, 160
Hydroecle of Neck, 112
Hyperthropy of Heart and Appoplexy, 578

I

Iodine in Glossitis, 140
Used Externally, 133
Impotence and Sterility, 41
Incarcerated Hernia, 567
Incontinence of Urine, 612
Indian Doctoring, 500
Indigo in Epilepsy, 433
Infanticide, 72
Inflamation of Testicles, 440
Inguinal Aneurism, 308
Introsusception, 145

J

Jurisprudence and Toxicology, 273

L

Labors Requiring Instruments, 582
Laryngitis, 596
Oil of Croton in 597
Lead in Tibia, 277
Lectures in Cincinnati College, 167
Ligature of Arteries in Aneurism, 421
Luxations of Clavicle, 272

M

Magnetism in Gout, 581
Malaria, 602
Medicine, 426
and Medical Jurisprudence, 430
in Denmark, 449
Medical Department of Cincinnati College, 623
Instruction, 480
in Paris, 132
Institutions in Paris, 96
Jurisprudence, 32
Students, 297
Method of Tying Divided Arteries, 634
Mental Philosophy, 324
Meteorological Observations, 640

PAGE
Milk Secretion Suspended, 332
Morphi in Burns, 505

N

Nervous Irritability, 169
Neuralgia, 598
Notices of Weather, Diseases, etc. 641
Nux Vomica on Animals, 261
Nymphomia, 105

O

Observations for 1837, 476
Our periodical, 166
Oxalic Acid, 447

P

Pathology and Practice of Physic, 208
Pectoral Disease, case of 201
Phthisis, By M. Louis, 173
Phthisis Pulmonalis, 527
Physical Signs of Disease, 369
Physiology of Vomiting, 605
Poisoning with Arsenic, 121
Cured by Hyd. Perox. Fer. 268, 466, 585
Poisons, Effects of 119
Population and Sustenance, 584
Power of Retaining Thigh in Socket, 425
Practice of Midwifery in Burmah, 140
Pregnancy, 62
Premature Birth of the Cord, 106
Professor Parker, 166, 629
Progress of Quackery, 644
Pulse Respiration and Temperature, 434
Prolapsus Uteri, 583
Puerperal Convulsions, 253
Purulent Matastasis, 267
Pylorus, Ulcers of 274

Q

Quack Doctors, 613, 619
Quackery, 296

R

Rape, 53
Remarkable Monstrosity, 280
Rheumatic Calarrhal Epidemic, 26
Rheumatism, Pathology of 262
Royal Academy of Medicine, 173

S

Scald Head, 271
Scalding by Steam, 478
Sequestra Removed, 118
Signs of Virginity, 56
Sleeplessness, 283
Spina Bifida cured, 631
Spinal Irritation, 513
Steam and Botanic Journals, 310
Sugar Detected in Blood, 446
Suicide in Geneva, 444

T

Tartar Emetic in Abstt. Practice, 161

PAGE

Temperature of Sinapisms, 268
Therapeutics, 257
Thorax changed by Age, 258
Tonic and Antidyspeptic Pills, 618
Traumatic Opthalmia, 572
Traveling Memoranda, 311, 469
Turpentine in Sciatica, 267
Typhus in the Young, 147

U

Urethera, Sore of 158

PAGE

V

Vaccination, 149
in France, 437
Variola, 149
Varioloid, 149
Varicocele, cure of 117
Venesection in Intermittents, 634

W

Wounded Intestines, 481

